# Postural orthostatic tachycardia syndrome in adolescents with ME/CFS — a case control study

**DOI:** 10.3389/fped.2026.1836407

**Published:** 2026-09-01

**Authors:** Ariane Leone, Katrin Gerrer, Anja Viereck, Annika Grabbe, Alissa Kircher, Uta Behrends, Andrea Maier

**Affiliations:** 1 MRI Chronic Fatigue Center for Young People (MCFC), Children’s Hospital, School of Medicine and Health, Technical University of Munich, Munich, Germany; 2 German Center for Infection Research, Munich Partner Site, Munich, Germany; 3 Department of Neurology, Medical Faculty RWTH Aachen University, Aachen, Germany

**Keywords:** adolescents, CFS, chronic fatigue syndrome, ME/CFS, orthostatic intolerance, postural orthostatic tachycardia syndrome, POTS, PoTS

## Abstract

**Introduction:**

Postural orthostatic tachycardia syndrome (PoTS) can be associated with myalgic encephalomyelitis/ chronic fatigue syndrome (ME/CFS). Pediatric PoTS requires a “sustained” rise in orthostatic heart rate ≥40 bpm above supine heart rate (relative limit), often >120 bpm (absolute limit). However, the diagnostic criteria for PoTS in adolescents remain underexplored, particularly regarding the definition of “sustained” tachycardia, which poses challenges in clinical and research settings. This study examined orthostatic intolerance criteria, including PoTS, in adolescents with ME/CFS and healthy controls.

**Methods:**

Medical history of orthostatic intolerance was assessed by a semi-structured interview with 18 ME/CFS patients and 18 matched healthy controls (14–17 years). A passive 10-min standing test with minute-by-minute heart rate and blood pressure registration was performed. PoTS was diagnosed by a clinical expert based on current consensus criteria. Data were analyzed using Fisher's exact test and receiver operating characteristic analysis.

**Results:**

Although history of orthostatic intolerance was positive in 15/18 ME/CFS [83%, 0.95 CI (61; 94)] and 3/18 healthy controls [17%, 0.95 CI (5.9; 39)], PoTS was diagnosed in only 7/18 ME/CFS [39%, 0.95 CI (20; 61)] vs. no healthy controls [0%, 0.95 CI (0; 18)]. Subgroups were identified, e.g., positive history of orthostatic intolerance yet physiological passive 10-min standing test, or negative history of orthostatic intolerance yet tachycardia in passive 10-minute standing test. PoTS diagnosis by the clinical expert matched best with the passive 10-min standing test alone when at least 60% of upright heart rate values were above one or both limits. The absolute limit was superior to the relative limit in distinguishing adolescents with and without PoTS.

**Discussion:**

PoTS was observed only in ME/CFS. In adolescents with ME/CFS, positive history of orthostatic intolerance, along with at least 60% of heart rate values above published upright-position limits, may allow non-experts to make a valid diagnosis, facilitating clinical care and future studies. Further research will show if these results can be generalized.

## 1 Introduction

Postural orthostatic tachycardia syndrome (PoTS, ICD-10 Germany G90.80, USA G90.A, ICD-11 8D89.2) in adolescents (12–19 years) is defined by symptoms of chronic orthostatic intolerance (OI) and a “sustained” heart rate (HR) increase in the upright position of at least 40 beats per minute (bpm) compared to the supine HR (1), or a standing HR of more than 120 bpm (2), in the absence of orthostatic hypotension (OH) or inappropriate sinus tachycardia (IST). HR is evaluated by a standing test or tilt table testing (TTT) (1). PoTS is often triggered by viral infections, shows a female predominance (3), and can be associated with myalgic encephalomyelitis/ chronic fatigue syndrome (ME/CFS, ICD-10-GM G93.3, ICD-10-CM G93.32, ICD-11 8E49) (1). ME/CFS is a multisystem chronic condition. In pediatric patients, it is characterized by impaired daily function with fatigue, post-exertional malaise (PEM), and unrefreshing sleep as cardinal symptoms for at least 3 months. Pediatric ME/CFS prevalence was estimated at 0.1%–0.9% worldwide (4, 5). Due to post-acute sequelae of coronavirus disease 2019, ME/CFS cases are increasing (6, 7). Though ME/CFS patients often report OI (4), data on prevalence of OI and PoTS in pediatric ME/CFS are conflicting due to small sample sizes, methodological disparities (8–11), and changes in the definitions of PoTS and ME/CFS over the last years (1, 4, 12). As a precise definition of a “sustained” HR increase is missing, the exact PoTS prevalence remains unclear. Moreover, the current PoTS definition does not explicitly address syndromes of OI with debatable transient or nonspecific tachycardia, nor diagnostics in the context of comorbidities (13, 14). In summary, a lack of consensus and clarity leads to a diagnostic dilemma that adversely affects patient care and clinical research (13, 15, 16).

Consequently, there is a pressing need for a standardized approach to diagnosing PoTS and OI syndromes (3, 17). Recent attempts to define “sustained” HR increases have been proposed (14). However, data on their overall validity are insufficient, particularly in adolescents exhibiting high HR variability and individuals with comorbidities. Therefore, this study addresses the following unresolved questions:

1. Do ME/CFS patients differ from Healthy Controls (HC) regarding their orthostatic HR and symptoms?
2. Are syndromes of OI, especially PoTS, more common in ME/CFS than in HC?
3. How can various syndromes of OI be differentiated from one another?
4. Can “sustained” HR increases be further specified by reliable cut-off values in a passive 10-min standing test (PST), to facilitate a PoTS diagnosis by non-experts?

By providing initial answers to these questions in our pilot study, we aim to contribute to a deeper understanding of pediatric PoTS with ME/CFS and provide a reproducible direction for future studies and clinical practice.

## 2 Methods

### 2.1 Ethics and data assessment

This study was approved by the local ethics committee (No. 618/21). Recruitment, informed consent, study visits, and data analyses were performed at a pediatric tertiary center specializing in ME/CFS. All data were processed via Microsoft® Excel® for Microsoft 365 MSO (Version 2310 Build 16.0.16924.20054) and R (Version 4.3.0, The R Foundation for Statistical Computing, Vienna, Austria, 2023) (18).

**Figure 1:**
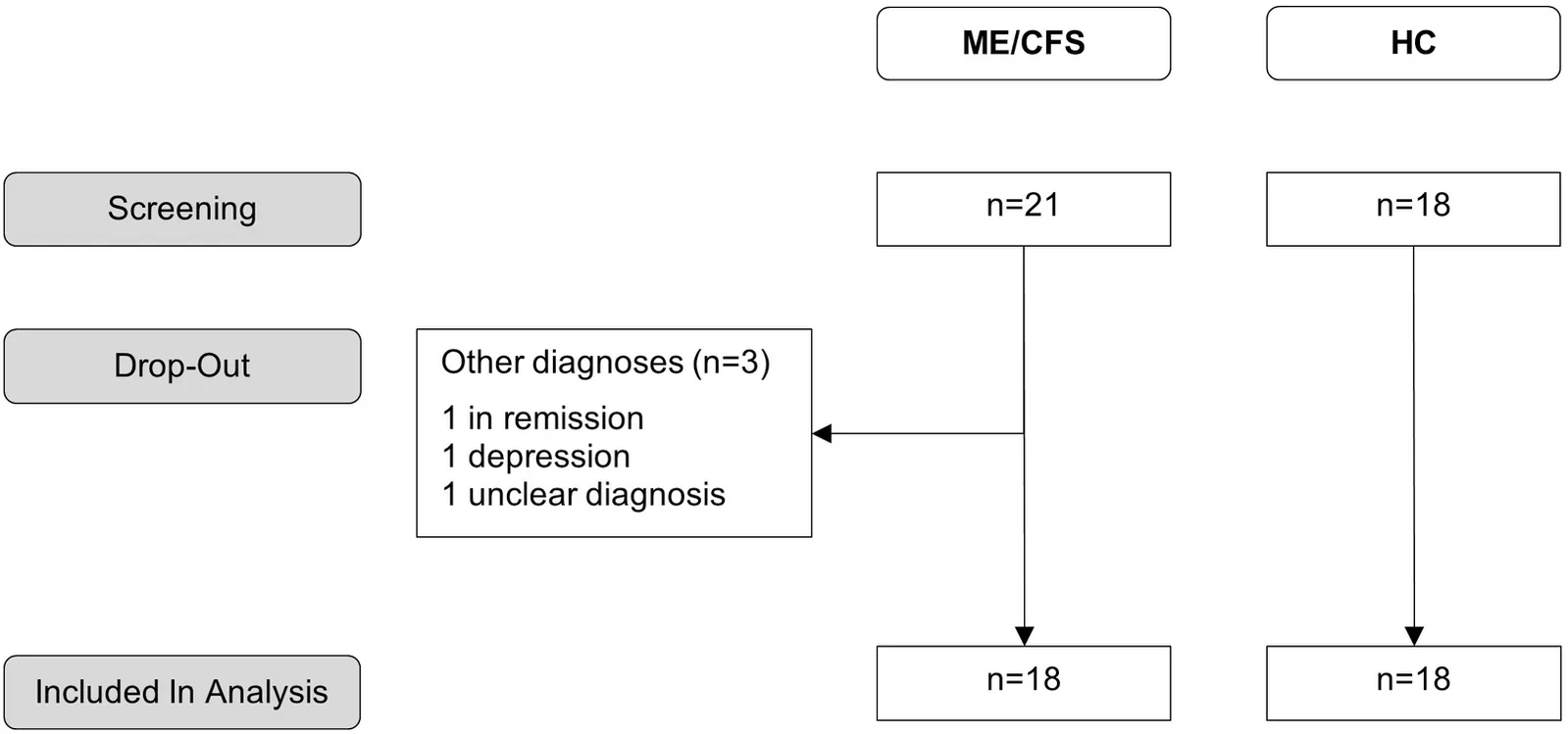
Recruitment process. *N* = 39 subjects were recruited, *n* = 21 for ME/CFS and *n* = 18 HC (Healthy Controls). In the ME/CFS-group, 3 subjects were not included in the final analysis because ME/CFS-diagnosis could not be confirmed during the study visit. In total, *n* = 36 subjects (*n* = 18 ME/CFS, *n* = 18 HC) were analyzed.

Data were evaluated in collaboration with a clinical expert (CE) specializing in autonomic neuropathies with 11 years of experience in diagnosing, treating, and investigating PoTS. ME/CFS patients (14–17 years) were compared to age- and sex-matched HC (Figure 1).

### 2.2 ME/CFS-diagnosis

ME/CFS was diagnosed using the Munich Berlin Symptom Questionnaire (19), which provides four PEM-based ME/CFS case definitions: the Canadian Consensus Criteria (20), the criteria by the Institute of Medicine (IOM) (8), and two pediatric case definitions by Jason et al. (5) and Rowe et al. (4). At least one of these definitions had to be met for the diagnosis of ME/CFS. Exclusion criteria for HC were any acute or chronic disease and any medication. Exclusion criteria for both groups were any contraindication for the PST (e.g., cardiac failure, severe aortic stenosis, inability to stand), pregnancy, and breastfeeding. Demographic parameters were assessed, including puberty status [status quo method (21)].

### 2.3 PoTS and OI diagnosis

**Figure 2:**
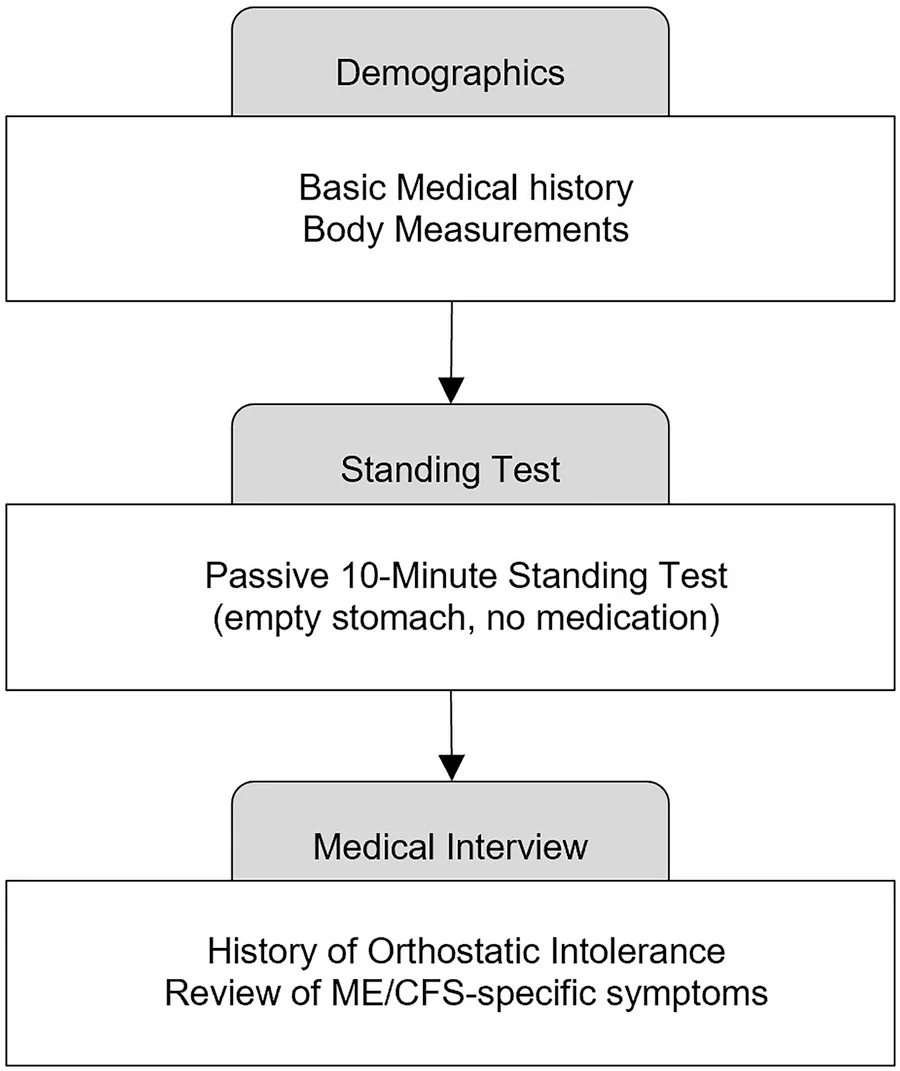
Study protocol. All study visits took place in the morning on empty stomach without morning medication. After taking basic medical history to confirm inclusion and exclusion criteria and basic body measurements, a passive 10-min standing test (PST) was performed, followed by the orthostatic interview.

Regarding the PoTS diagnosis, we performed a PST and conducted a structured orthostatic interview. The PST was performed under standardized conditions (in the morning, on an empty stomach, without morning medication) (Figure 2). PST was used instead of the active stand test or TTT. This choice was made to provide an accessible, widely available alternative to TTT because TTT—considered the gold standard for diagnosing POTS with continuous blood pressure and HR measurement—is often unavailable in clinical care and at our primary study center for this research. PST has been validated as a measure of OI (22, 23) and has been used previously in ME/CFS (24). For practical clinical implementation, approximately 30 min should be allocated for testing, including a period of quiet supine rest, a 10-min active standing phase, and the time required for preparation and documentation. The PST protocol was similar to the NASA Lean Test (22, 24–26), with minor differences (empty stomach, 5-min supine HR measurements before and after standing). HR, Blood Pressure (BP), and orthostatic symptoms were non-invasively assessed every minute using the CARESCAPE V100 (GE Healthcare). After 5 min of lying supine, the measurement started. After 5 more minutes supine, participants stood up and leaned with their shoulders against a wall for up to 10 min, their feet 15 cm apart from the wall (passive standing). This approach allowed an upright position of about 70°, as used in the TTT (27, 28). Standing was followed by another 5 min of lying. The standing period was shortened if participants experienced unbearable symptoms. Stopping criteria were defined as the inability to continue standing due to any newly arising, intolerable symptoms and/or a sustained HR > 200/min for 2 min for safety reasons.

History of OI (HOI) was assessed by a study physician in a medical interview. HOI included 18 predefined symptoms like palpitations, dizziness, nausea, and presyncope. The participants were free to name new symptoms. The outcome was categorized later in the discussion with the CE (blinded data presentation) as **positive** or **negative HOI**. A positive HOI was determined by clinically significant symptoms for at least 3 months that are endured while standing and improved with recumbence (1, 2).

The data were presented to the expert in a blinded, stepwise manner:

1. **HR Curve Analysis:** The expert had to evaluate the PST HR curve alone, without any information about the patient's symptoms. The expert classified the curve as either PoTS-like or not. By not being aware of orthostatic symptoms, the expert could not be biased towards or against diagnosing definite PoTS.
2. **Orthostatic Symptom History:** In the second step, the results of the detailed orthostatic interview were provided, addressing the frequency and duration of orthostatic symptoms.

**Figure 3:**
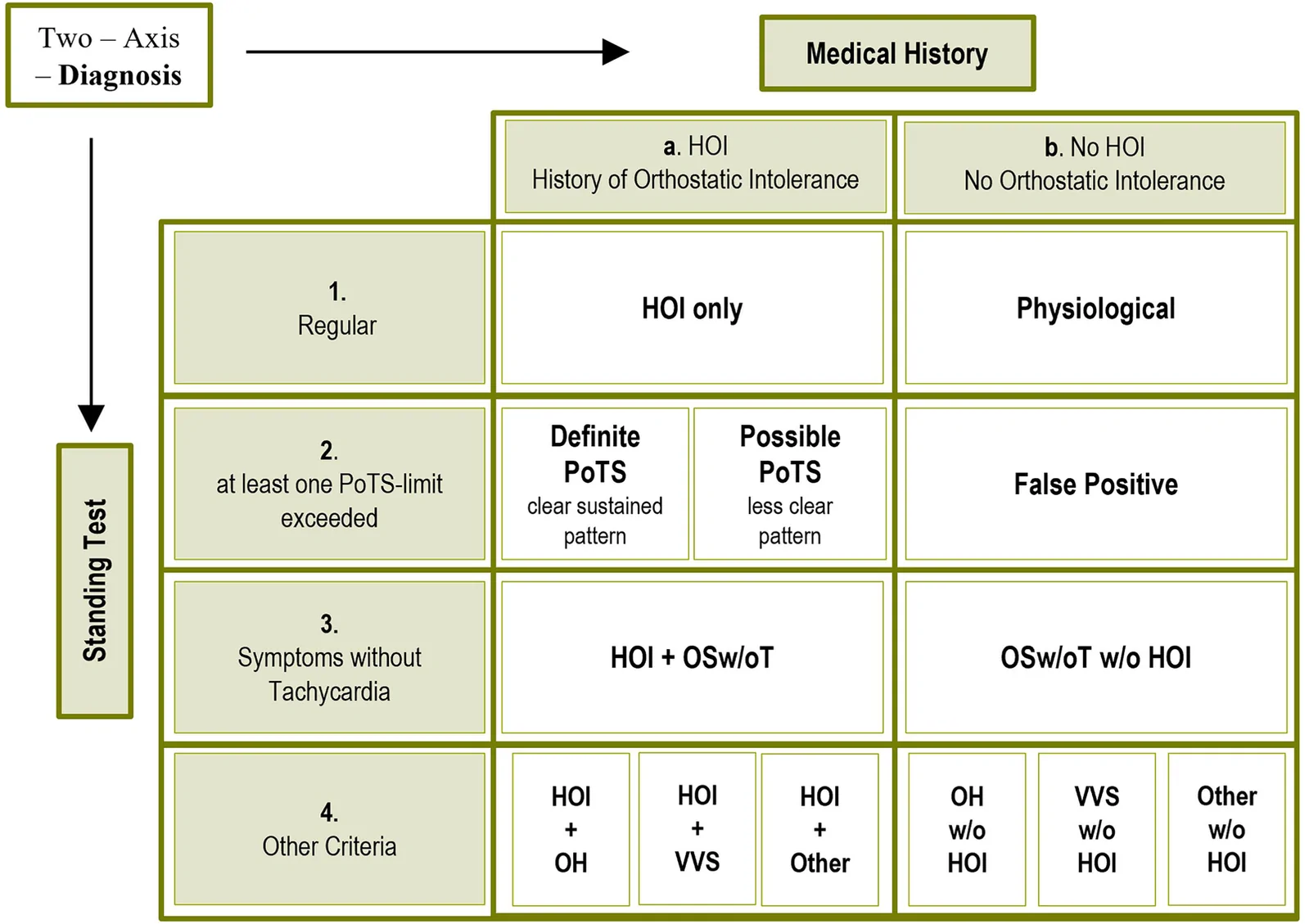
Orthostatic diagnostic workflow. Orthostatic diagnoses are defined by the combination of the Medical History of Orthostatic Intolerance (HOI, *x*-axis) and the passive 10-min standing test (PST, *y*-axis). The HOI interview had two outcomes: a = positive history, b = negative (no history of OI). The PST had four outcomes: 1. No circulatory disorder [regular heart rate (HR) and blood pressure (BP) curve], 2. HR elevated (sustained above the limits or above the limits but not sustained, without sustained BP-drop), 3. symptoms without tachycardia (symptoms during PST but no pathological HR-elevation), 4. other criteria (HR and BP show other abnormality). HOI positive diagnoses included 1a) HOI only (during PST regular HR and no symptoms, HOI positive), 2a1) definite PoTS (during PST HR persistently elevated, HOI positive), 2a2) possible PoTS [during PST HR elevated, but not fulfilling the criteria of “sustained” in the opinion of the clinical expert (CE), HOI positive], 3a) HOI + OSw/oT (orthostatic symptoms during standing without tachycardia, no pathological HR-elevation, HOI positive), 4a) HOI+ specific diagnosis (HR and BP fulfilled other criteria, e.g., OH, HOI positive). HOI negative diagnoses included 1b) physiological status (during PST regular HR and no symptoms, HOI negative) 2b) false positive (during standing HR persistently or not persistently elevated without symptoms, HOI negative), 3b) OSw/oT w/o HOI (orthostatic symptoms during standing without tachycardia, HOI negative), 4b) specific diagnosis w/o HOI (HR and BP fulfilled other criteria, e.g., OH, HOI negative).

Based on this procedure, the final diagnosis of definite PoTS was defined, considering the established diagnostic criteria: Positive HOI for at least 3 months combined with a sustained relative HR increase of at least 40 bpm (1) or an absolute HR of more than 120 bpm (2) while standing, in the absence of OH or IST. A sustained heart increase was judged by the CE's expertise based on the visual assessment of the HR curve within an orthostatic diagnostic structure (Figure 3). A sustained HR increase (=PoTS-like curve) was defined as an elevation of ≥40 bpm or 120 bpm that persisted over the majority of the upright phase and presented as a stable, plateau-like elevation rather than isolated or short-lived peaks, as judged by the CE.

- If the history of orthostatic intolerance (HOI) aligned with a POTS-like HR curve, a diagnosis of POTS was made.
- If no orthostatic symptoms were reported, but the HR curve was judged POTS-like, the case was classified as “false positive.”
- Conversely, if HOI was present but the HR curve was normal, it was classified as HOI without POTS.
- Participants who showed orthostatic symptoms during the PST, without a POTS-like curve, were classified as HOI with orthostatic symptoms without tachycardia, or as orthostatic symptoms without HOI if the history was negative.
- Cases of OH or possible vasovagal syncope were categorized as “Other.”

OH is defined as a sustained decrease in systolic BP by 20 mmHg or diastolic BP by 10 mmHg (29–31).

We also distinguished between “definite PoTS” and “possible PoTS”. Both require HOI. “Definite PoTS” additionally required the sustained HR pattern. “Possible PoTS” was assigned in borderline heart pattern constellations, in which a ≥40 bpm or >120 bpm increase was observed, but the overall pattern was less clearly sustained (e.g., fluctuating or only intermittently elevated values without a convincing plateau).

### 2.4 Analysis of HR values

The frequency distribution of all possible eight orthostatic diagnoses in both groups (ME/CFS vs. HC) was analyzed using Fisher's exact test.

HR values were calculated as the arithmetic mean for every minute during the PST with a 95% confidence interval (CI). The relative HR limit was calculated as the sum of the mean supine HR (over the first 5 min of lying) plus 40 bpm. The relative and absolute HR limits were analyzed using a Receiver Operating Characteristic (ROC) curve to determine the optimal cut-off for an objective, definite diagnosis of PoTS across the whole sample (ME/CFS and HC). The CE's final diagnosis was defined as the gold standard. As a cut-off criterion, we defined the number of HR values above the published HR limits for each PST, divided by the total number of HR values measured in that PST, yielding the percentage of elevated HR values in each PST. This strategy allowed the inclusion of shorter PSTs, which had to be stopped due to severe symptoms of OI. The final analysis included HR values from minutes two to ten during standing. The first minute of standing was excluded because it took less mobile patients longer to assume a standing position. Cases were defined as “definite PoTS” and were compared to all other orthostatic diagnoses. The true positive rate (sensitivity) was compared with the false positive rate (1-specificity). The optimal threshold was determined by the optimization criteria $y=\min({\left(1-\mathrm{sensitivity}\right)}^{2}+{\left(1-\mathrm{specificity}\right)}^{2})$, yielding the highest sensitivity with the lowest possible false positive error (32).

## 3 Results

### 3.1 Demographics

Out of 21 ME/CFS patients screened, 18 (6 males, mean age 15.4 ± 1.0 years, median disease duration 19 months, mean absolute deviation 17,7 months) were included in the final analysis and matched to 18 HC (6 males, mean age 15.1 ± 1.1 years) (Figure 1). Both groups showed no significant differences in age, biological sex, body mass index, or pubertal development (Table 1). 4/18 (22.2%) of ME/CFS patients have been diagnosed with a post-COVID condition (ICD-10 GM U09.9). No participant was receiving medication known to significantly modulate autonomic function (e.g., vasoactive drugs). 17/18 (94.4%) patients with ME/CFS reported using nutritional supplements, such as vitamins or fatty acids. Due to the limited sample size, further subgroup analyses were not feasible.

**Table 1:** Demographic characteristics.

Characteristic	Total *n* = 36	ME/CFS *n* = 18	HC *n* = 18	*p*-value
Age	15.3 ± 1.1	15.4 ± 1	15.1 ± 1.1	0.35
Sex (biological)				1.00
Male	12 (33.3%)	6 (33.3%)	6 (33.3%)	
Female	24 (66.7%)	12 (66.7%)	12 (66.7%)	
BMI (kg/m^2^)	20.9 ± 2.9	21.5 ± 2.7	20.3 ± 2.5	0.18
Puberty development				1.00
(post)pubertal	11 m (91.7%) 24 w (100%)	5 m (83.3%) 12 f (100%)	6 m (100%) 12 f (100%)	
Prepubertal	1 m (8.3%) 0 f (0.0%)	1 m (16.7%) 0 f (0.0%)	0 m (0.0%) 0 f (0.0%)	
Additional Diagnoses				
Diabetes mellitus type 1	1	1 (5.6%)	–	–
Post-COVID	4	4 (22.2%)	–	–
Migraine with aura	1	1 (5.6%)	–	–

ME/CFS vs. healthy controls (HC).

### 3.2 Orthostatic analysis in ME/CFS vs. HC

**Figure 4:**
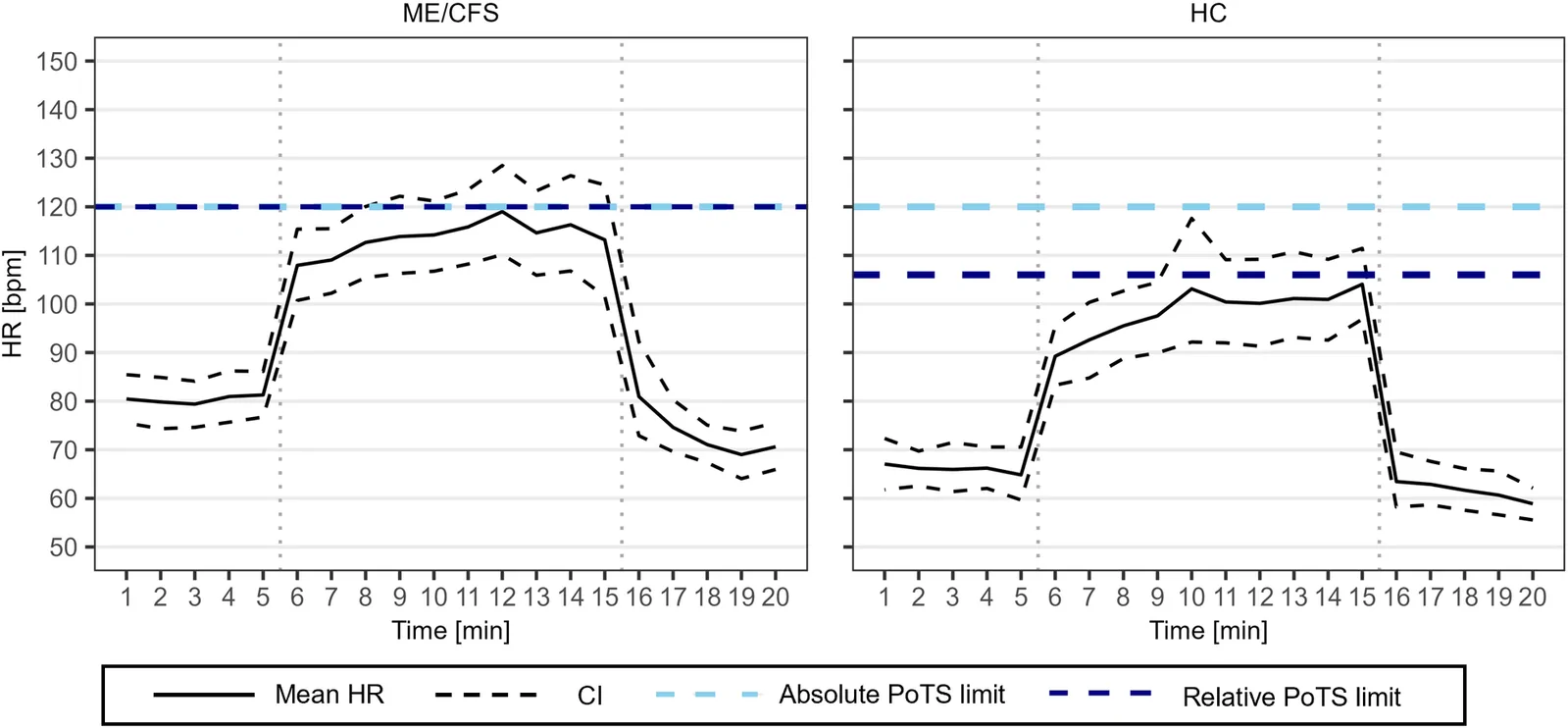
Mean heart rates (HR) with confidence interval (CI) during passive 10-minute standing test (PST) for ME/CFS patients and healthy controls (HC). Vertical dotted lines indicate a change in posture shortly after minutes 5 (supine to standing) and 15 (standing to supine). 8/18 ME/CFS and 2/18 HC had to stop the PST early, due to severe orthostatic intolerance symptoms.

Mean HR in ME/CFS was significantly higher than in HC in the supine (*p* = 1.0 × 10^−4^ < 0.001) and the standing position (*p* = 0.0059 < 0.01) (Table 2). Figure 4 shows the mean HR curves for ME/CFS and HC, with CIs. The threshold for PoTS in the ME/CFS group was consistent, with the relative limit (at least 40 bpm increase above the supine mean HR) and the absolute limit (120 bpm) aligning. For HC, the relative limit was 106 bpm. The mean HR curves of both groups did not exceed any PoTS limit. 8/18 ME/CFS and 2/18 HC had to stop the PST early due to severe OI symptoms. On average, test cancellation occurred after 5.4 ± 1.6 min of standing. BP was steady except in one case, which could be classified as OH. No single symptom alone led to test termination; rather, the combination and intensification of multiple symptoms resulted in the inability to continue standing. All terminated tests were associated with at least two concurrent symptoms. Among these, dizziness was the most frequently reported symptom (90% of participants who stopped), followed by headache (60%), muscle weakness/tremor/pain (40%), nausea (30%), heart palpitations (30%), and tingling 20%). Less frequently reported symptoms included presyncope, dyspnea, sensations of heat or cold, visual disturbances, sweating and fatigue (each 10%).

**Table 2:** HR during PST.

Heart rate (bpm)	ME/CFS *n* = 18	HC *n* = 18	*p*-value
Supine	80.4 ± 10.3	66.0 ± 9.2	0.0001
Passive standing	116.0 ± 15.4	100.0 ± 17.2	0.0059

Heart rate (mean ± standard deviation) in bpm. ME/CFS vs. healthy controls (HC).

The frequency distribution of orthostatic subtypes (Table 3, Figure 3) differed highly significantly between ME/CFS and HC (*p* = 2.71 × 10^−4^ < 0.001, Cramer's *V* = 0.76). A definite PoTS was diagnosed in 7/18 ME/CFS patients [39%, 0.95 CI (20; 61)] vs. no HC [0%, 0.95 CI (0; 18)]. One HC but no ME/CFS patients met the OH criteria, and two ME/CFS patients vs. one HC met the possible PoTS criteria. HOI was positive in 15/18 ME/CFS [83%, 0.95 CI (61; 94)] ME/CFS and 3/18 HC [17%, 0.95 CI (5.9; 39)].

**Table 3:** Frequency distribution of orthostatic diagnoses.

Orthostatic subgroup	ME/CFS *n* = 18	HC *n* = 18	Total *n* = 36
HOI only	0 (0.0%)	0 (0.0%)	0 (0.0%)
Physiological	1 (5.6%)	8 (44.4%)	9 (25.0%)
Definite PoTS	7 (38.9%)	0 (0.0%)	7 (19.4%)
Possible PoTS	2 (11.1%)	1 (5.6%)	3 (8.3%)
False Positive	1 (5.6%)	6 (33.3%)	7 (19.4%)
HOI + OSw/oT	6 (33.3%)	1 (5.6%)	7 (19.4%)
OSw/oT w/o HOI	1 (5.6%)	0 (0.0%)	1 (2.8%)
HOI + OH	0 (0.0%)	1 (5.6%)	1 (2.8%)
HOI + VVS	0 (0.0%)	0 (0.0%)	0 (0.0%)
HOI + Other	0 (0.0%	0 (0.0%)	0 (0.0%)
OH w/o HOI	0 (0.0%)	0 (0.0%)	0 (0.0%)
VVS w/o HOI	0 (0.0%)	0 (0.0%)	0 (0.0%)
Other w/o HOI	0 (0.0%)	1 (5.6%)	1 (2.8%)

Counts given in total and by groups, MECFS vs. healthy controls (HC). Orthostatic Subgroups are defined by History of Orthostatic Intolerance (HOI) and the passive 10-min standing test (PST) (see Figure 3).

**Figure 5:**
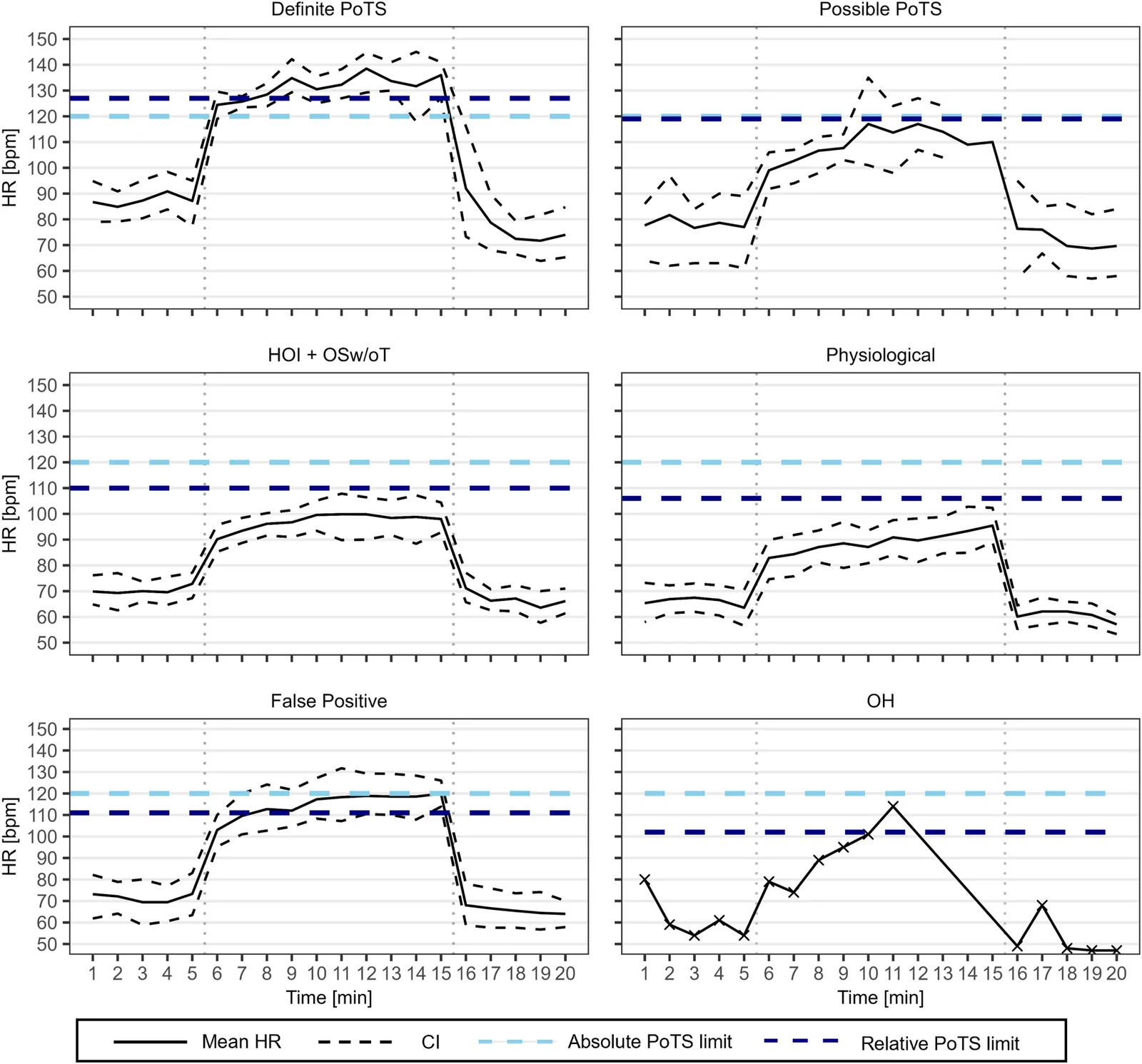
Mean heart rates (HR) during passive 10-minute standing test (PST) for orthostatic subgroups. Vertical dotted lines indicate a change in posture shortly after minutes 5 (supine to standing) and 15 (standing to supine). The confidence interval (CI) could not be calculated for all data points as several participants had to stop due to strong orthostatic symptoms. For orthostatic subgroups see Figure 3 or Table 4.

Comparing the mean HRs during standing between the different orthostatic subgroups reveals the highest HRs in the group with definite PoTS, followed by false positives and possible PoTS (Figure 5, Table 4): Definite PoTS had not only the highest HR but also the highest relative PoTS limits (127 bpm). Participants with possible PoTS, physiological status, and other diagnoses did not exceed any PoTS limits. Only the upper CI of the possible PoTS subgroup exceeded the PoTS limits. The seven false positives included six HCs and one ME/CFS patient, with five HCs exceeding the relative PoTS limits and one HC and one ME/CFS patient exceeding the absolute limits. False positives exceeded the relative PoTS limit only in the second half of the standing period. BP stayed stable except for one case of Orthostatic Hypotension (see Supplementary Table S1).

**Table 4:** Overview of main characteristics and findings for each orthostatic subgroup.

Orthostatic Subgroup	HOI	PST	HR supine	HR standing	Relative Limit	Absolute Limit
HOI only	Yes	Regular	–	–	–	120
Physiological	No	Regular	65.5 ± 9.8	89.0 ± 12.4	105.4 ± 9.8	120
Definite PoTS	Yes	At least one PoTS-Limit exceeded, sustained HR pattern	87.4 ± 9.8	131.0 ± 4.5	127.3 ± 10.0	120
Possible PoTS	Yes	At least one PoTS-Limit exceeded, less clearly sustained HR pattern (e.g., fluctuating)	78.3 ± 13.6	110.4 ± 8.2	118.7 ± 13.6	120
False Positive	No	PoTS-Limit exceeded (sustained or unsustained)	71.5 ± 13.1	114.9 ± 11.9	111.4 ± 13.0	120
HOI + OSw/oT	Yes	Symptoms without Tachycardia	70.3 ± 7.2	97.2 ± 7.1	110.4 ± 7.3	120
OSw/oT w/o HOI	No	Symptoms without Tachycardia	(75)	(99.8)	(115)	120
HOI + OH	Yes	Orthostatic Hypotension	(61.6)	(92)	(102)	120
HOI + VVS	Yes	Vasovagal Syncope	–	–	–	120
HOI + Other	Yes	not identifiable	–	–	–	120
OH w/o HOI	No	Orthostatic Hypotension	–	–	–	120
VVS w/o HOI	No	Vasovagal Syncope	–	–	–	120
Other w/o HOI	No	Not identifiable	(66.2)	(118.2, sudden maximum 190)	(106)	120

Orthostatic Subgroups are defined by the history of Orthostatic Intolerance (HOI) and the passive 10-min standing test (PST) (see Figure 3). Heart rate (HR) in the supine and standing positions is shown as mean and standard deviation in bpm. The relative PoTS Limit is given, as well as the absolute PoTS Limit in comparison. In subgroups with count *n* = 1, the result is in brackets, as valid statistics are not possible here.

### 3.3 Comparison of PoTS criteria

**Figure 6:**
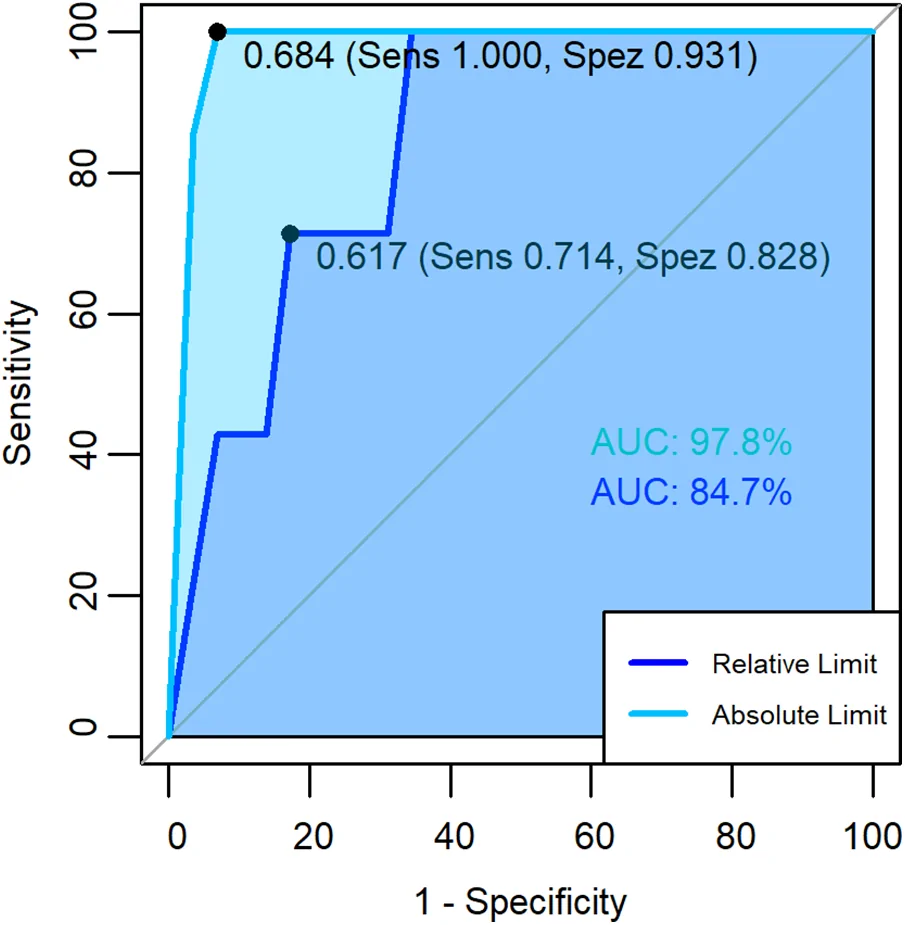
ROC-Analysis for relative and absolute PoTS-limits. The best cut-off for a diagnosis of definite PoTS was 62% for the relative PoTS limit and 68% for the absolute PoTS limit, with the cut-off defined as the percentage of elevated heart rate values during the 10-min passive standing test above the respective limit.

ROC analyses were performed for 0 to 100% HR values above both limits, comparing definite PoTS with all other diagnoses combined. Optimal threshold analyses yielded 61.7% for relative PoTS limits (sensitivity/specificity = 0.714/0.828) and 68.4% for absolute PoTS limits (sensitivity/specificity = 1.000/0.931). The AUC was 85% for the relative PoTS limit and 98% for the absolute PoTS limit (Figure 6).

## 4 Discussion

### 4.1 Key findings

This case-control study on adolescents with ME/CFS revealed four key findings: First, ME/CFS patients had significantly higher orthostatic and supine HRs than HCs. Secondly, syndromes of OI were significantly more common in ME/CFS than in HC. Thirdly, the syndromes of OI identified in this study were correlated with distinct HR patterns observed over a 10-min period of standing. Finally, a conclusive diagnosis of PoTS in this cohort was most effectively distinguished from other orthostatic diagnoses and physiological status when at least 60% of the HR values in the standing position exceeded the absolute or the relative limit.

### 4.2 Limitations

Limitations of this pilot study must be considered when interpreting the results. The low sample size limits the generalizability of our findings. A second limitation is the potential bias arising from the single-center design. Recruiting bias may have occurred, as some volunteers were informed by participants. The explicit call for “healthy” participants might have discouraged adolescents with OI from participating. Given the exploratory nature of this study, our findings require validation in larger, multicenter cohorts. Larger sample sizes would enable more detailed subanalyses, including comparisons of ME/CFS patients with and without post-COVID-19 conditions, as well as analyses stratified by disease duration and medication profiles. As essential strengths, we used strict PEM-based criteria for ME/CFS diagnosis, selected study participants within a narrow age range to minimize age-dependent variations in circulatory parameters (33–35), and applied a thorough subgroup analysis of OI syndromes.

### 4.3 Orthostatic analysis in ME/CFS vs. HC

In line with our study, other groups found higher standing HRs in ME/CFS than HC (11, 16, 36). Yet, in our ME/CFS patients, HRs were higher during standing and supine positions compared to HC (Figure 4), possibly indicating a general sympathetic overactivation in ME/CFS (1, 17). This finding might be explained by the selection of severely ill patients during medical visits based on strict, PEM-based ME/CFS criteria, compared to earlier studies that used the polythetic Fukuda criteria (11, 37) or screened only by telephone interviews without further medical evaluation (36). As the mean supine HR of ME/CFS was higher than that of HC, their relative PoTS limits were also higher. However, a remarkable statistical spread of HR values was evident across both groups, as shown by the CI limits (Figure 4). This spread could be traced back to the underlying specific orthostatic subgroups (Figure 5, Table 3). Interestingly, systolic BP was also higher in ME/CFS than in HC (Supplementary Figures S1–S3), again suggesting a sympathetic overactivation or reduced parasympathetic activation in ME/CFS (38, 39). For adolescents with ME/CFS, elevated nocturnal BP compared to HC (40) and altered BP variability have been reported (41).

In our study, orthostatic diagnoses were significantly more frequent in ME/CFS patients than in HCs. Since autonomic dysregulation plays a major role in the clinical picture of ME/CFS, this finding was not unexpected. An association between ME/CFS and OI has been described in several studies and is included in the IOM diagnostic criteria (4, 8). However, some studies do not report a higher prevalence of PoTS/OI in ME/CFS than in HC (9, 36). We attribute our findings to the detailed OI-specific interview, which minimized the risk of overlooking orthostatic symptoms, and the strict, PEM-based ME/CFS diagnosis, which minimized the risk of including other diseases that might have been presented with fatigue.

Experts in this field have postulated the existence of orthostatic subgroups in adults, based on symptoms and hemodynamic criteria similar to those in our study (Figure 3) (1, 14). We appreciate this approach, as it aligns with our clinical observations in OI. Yet, published data are insufficient to reliably identify orthostatic subgroups in adolescents (14). To our knowledge, our study provides, for the first time, data on orthostatic subgroups in adolescents with ME/CFS and age-matched HC (Figure 5).

The final PoTS diagnosis by the CE was based on 11 years of specialized experience with TTT, using the criterion of a “sustained” HR increase. The CE distinguished “definite POTS” from “possible POTS” when a PoTS could not be clearly diagnosed nor excluded. This very strict approach to PoTS diagnosis might explain why we found a lower frequency of definitive PoTS among ME/CFS patients in this study than in previous studies by our group (19, 42).

The orthostatic subgroups included “postural symptoms without tachycardia” (OSw/oT + HOI), a condition frequently encountered in clinical practice and recently addressed by the Canadian Cardiovascular Society (14). Knowledge of its pathophysiology is still limited (14). This orthostatic subgroup might represent a preliminary stage of PoTS, longstanding PoTS under therapy, or another functional autonomic disorder. To date, PoTS and OI are defined by hemodynamic criteria and symptoms, yet other causes and etiologies are discussed (43). Notably, most orthostatic diagnoses were identified within the ME/CFS group (Table 3). It is plausible that ME/CFS may influence the HR itself, and this factor cannot be discounted.

### 4.4 Comparison of PoTS criteria

There is an ongoing debate about the PoTS thresholds for adolescents in PST and TTT. Boris et al. questioned the postulated higher relative limit for adolescents (+40 bpm) compared to adults (+30 bpm) because symptoms were identical with a 30 bpm or 40 bpm increase in HR (13). Singer et al. found a substantial overlap between HRs in HC and PoTS and therefore suggested that pediatric PoTS should be diagnosed only in cases with a relative orthostatic HR increase of 40 bpm and/or an absolute orthostatic HR ≥130 bpm (≤13 years) and ≥120 bpm (≥14 years) within 5 min of TTT (16). Our findings argue for both: On the one hand, groups with “OSw/oT + HOI” or “possible PoTS” exhibited significant symptom burdens despite only borderline HR increases, suggesting that the PoTS limits should be lowered. On the other hand, false-positive individuals mainly showed HR values above the relative PoTS limits, suggesting a potential need to raise the limits. Overall, orthostatic conditions in adolescents, given their high HR variability, might not be adequately captured by simple classifications of PoTS vs. OH vs. non-PoTS, but rather reflect a more complex situation. This complicates a clear case definition of PoTS in adolescence, especially for non-experts. We approached this question as suggested by Raj et al. (14) and asked whether the number of HR values above the PoTS limits might help differentiate definite PoTS from other syndromes of OI and physiological findings. The Canadian Cardiovascular Society suggested two HR values, at least 1 min apart, above the adults’ limits, but evidence for this approach is missing (14). Moreover, two HR values above PoTS limits are more significant in cases with a standing period of 4 min (50%) than in those with 10 min (20%), thus introducing a bias toward shorter periods. To overcome differences in standing periods across PSTs with ME/CFS, Lee et al. suggested evaluating the last 3 min of standing (24), resulting in a similar bias.

Instead of indicating a cut-off at an absolute number of increased HR values, our data suggested setting a relative cut-off at 60%–70% HR values above both standard PoTS limits (Figure 6). This approach allows inclusion of all standing lengths, and the result aligns with Singer et al.'s postulation of a strict definition (16). The sensitivity and specificity of our method meet and, in most cases, exceed those of the other proposed methods (Supplementary Tables S2, S3).

In summary, we suggest a PoTS case definition for adolescents with HR values above 60% in either of the two standard PoTS limits, combined with a positive HOI. To avoid false positives, we recommend an absolute limit of 120 bpm, as it yielded excellent specificity. Patients with OSw/oT + HOI or possible PoTS should be carefully reassessed since they carry a significant orthostatic symptom burden.

### 4.5 Conclusion

We report the results from a prospective case-control study on OI syndromes, including PoTS, in adolescent ME/CFS patients and present an approach to standardized PoTS diagnostics. Currently, there is no clear, data-driven definition for diagnosing POTS in children and adolescents, leaving significant variability in diagnostic practices. Our study addresses this critical gap by proposing a threshold: a sustained HR elevation of at least 60% of the total standing time, which has not been previously investigated in this detail.

Additionally, we identify the value of the absolute HR limit (orthostatic HR at least 120 bpm) in distinguishing false positives—a key contribution that provides a more precise framework for diagnosis. This approach could enhance diagnostic accuracy, reduce misclassification, and improve the identification of POTS cases in this population. In clinical practice, we suggest performing a medical interview addressing orthostatic symptoms and a passive 10-min standing test. Patients with other syndromes of OI should be reevaluated in follow-up visits, as a definite PoTS might develop. For future research, we plan to expand our two-step, blinded methodology in a larger study by involving multiple experts independently. Larger studies are needed to verify these findings and to finally establish a guideline for a standardized PoTS diagnosis by non-experts treating ME/CFS patients and others with compromising orthostatic symptoms.

## Data Availability

The datasets presented in this article are not readily available because Data Sharing can be only be requested for legitimate reasons. Requests to access the datasets should be directed to Ariane Leone, ariane.leone@tum.de.
