# Supplementary material for: Postural orthostatic tachycardia syndrome in adolescents with ME/CFS — a case control study
Source: Front Pediatr. 2026 Sep 1;14:1836407. doi: 10.3389/fped.2026.1836407 (PMC13574936; doi:10.3389/fped.2026.1836407)
Supplement: Supplementary file 1 [file Supplementaryfile1.pdf]

Supplementary Material

1 Supplementary Figures and Tables

1.1 Supplementary Figures

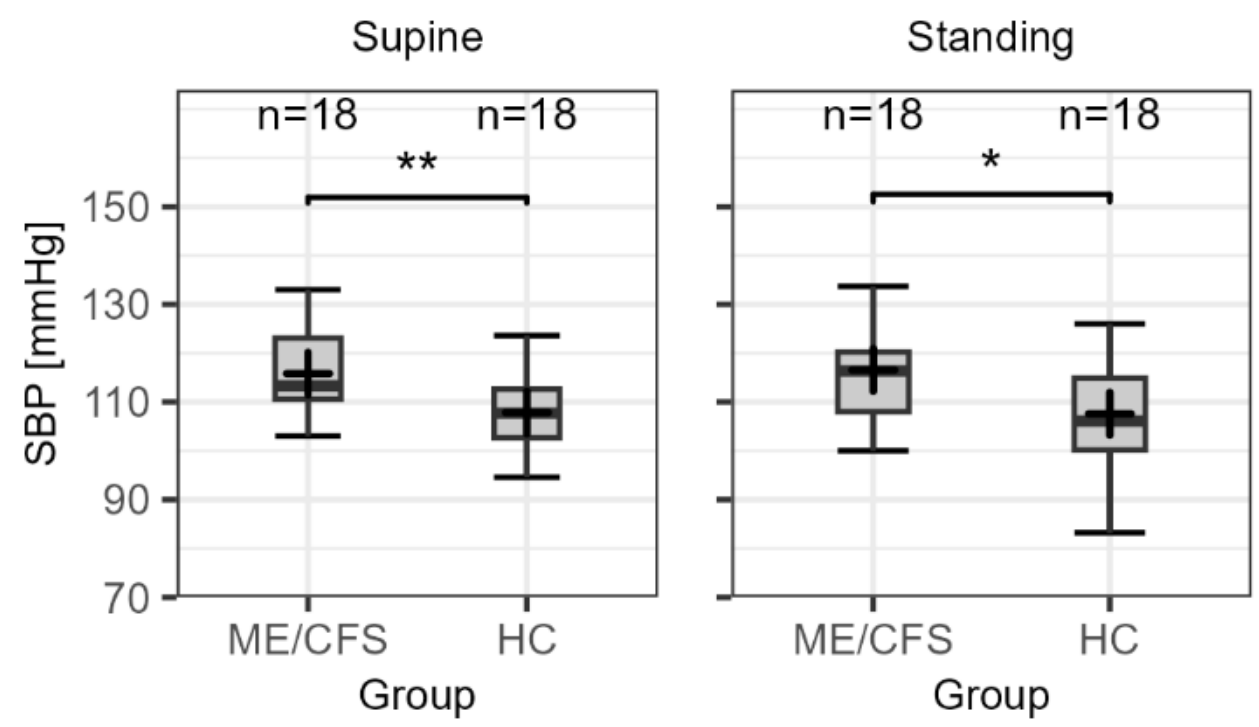

Supplementary Figure 1. Systolic Blood Pressure during PST for ME/CFS vs. HC.
Systolic Blood Pressure (SBP) is significantly higher in ME/CFS than in HC supine and upright. P-Value (t-test): * < .05, ** <.001. ***<.0001.

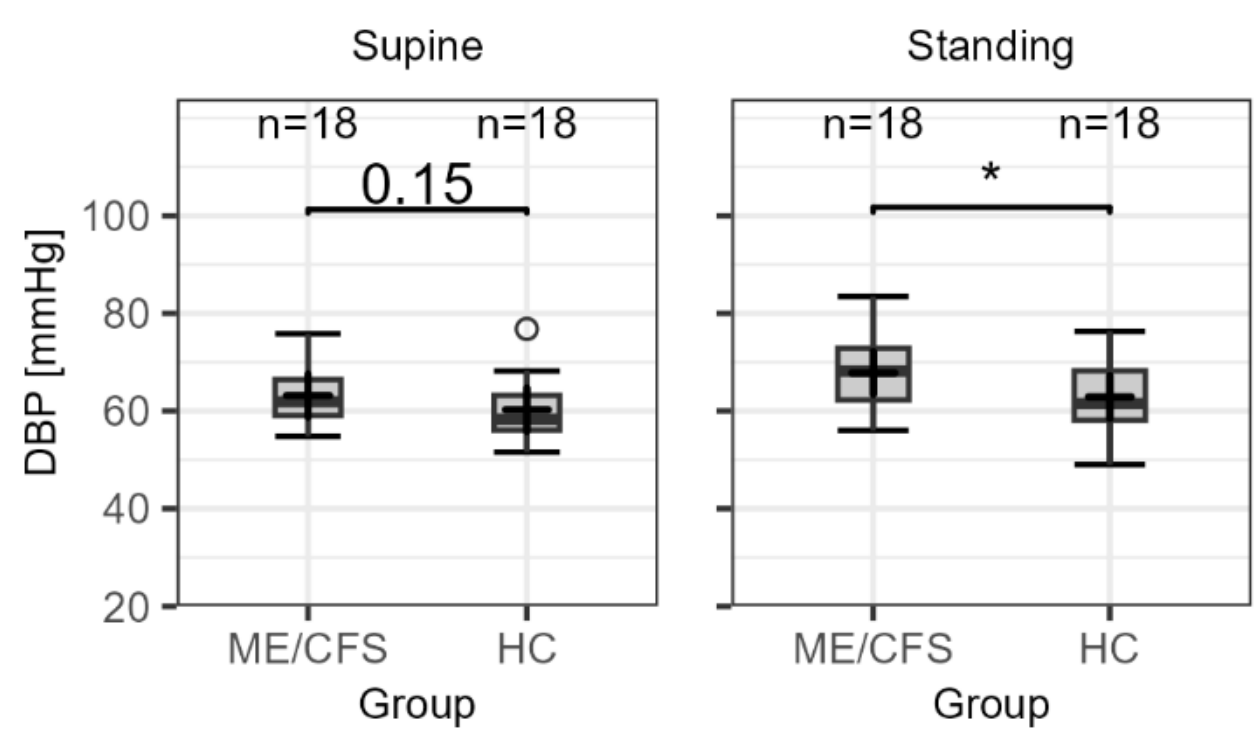

Supplementary Figure 2. Diastolic Blood Pressure during PST for ME/CFS vs. HC.
Diastolic Blood Pressure (DBP) is significantly higher in ME/CFS than in HC only upright, but not supine. P-Value (t-test): * < .05, ** <.001. ***<.0001

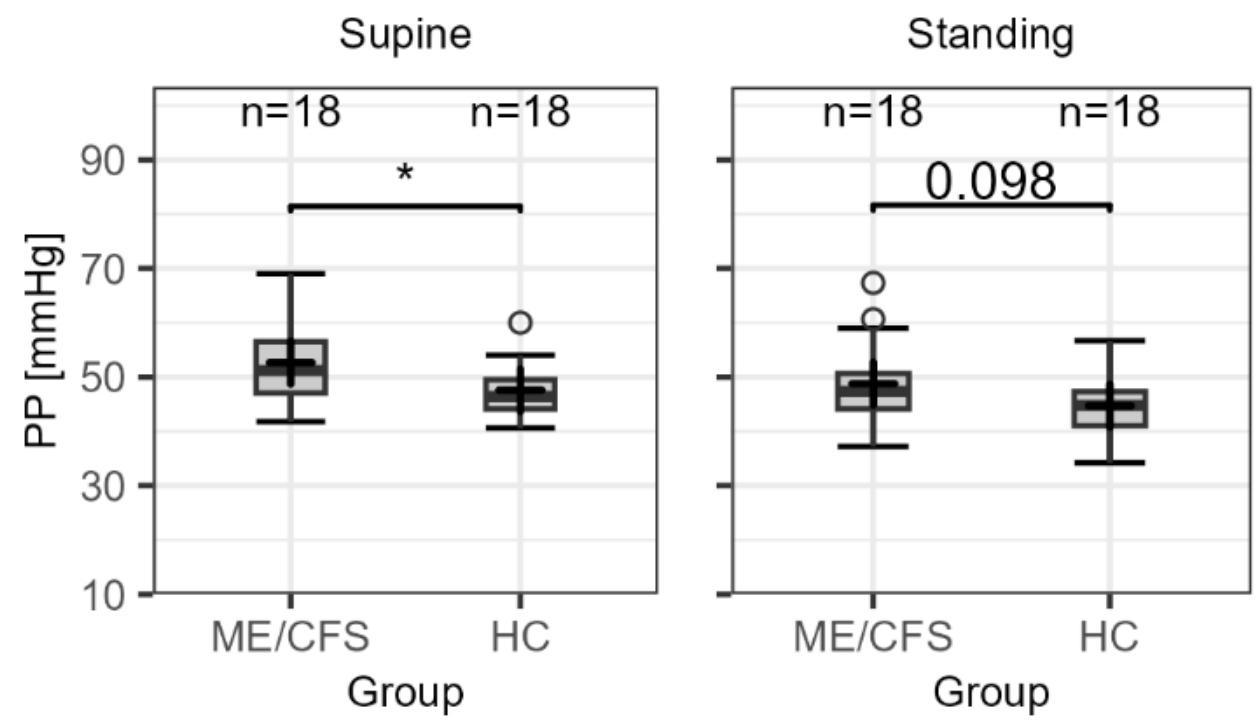

Supplementary Figure 3. Pulse Pressure during PST for ME/CFS vs. HC.
Pulse Pressure (PP) is significantly higher in ME/CFS than in HC only supine, not upright. P-Value (t-test): * < .05, ** <.001. ***<.0001

1.2 Supplementary Tables

Supplementary Table 1. Orthostatic Subgroups are defined by the history of Orthostatic Intolerance (HOI) and the passive 10-minute standing test (PST) (see Figure 3). Counts are given in total and by group (ME/CFS and Healthy Controls (HC)). Systolic Blood Pressure (SBP) and Diastolic Blood Pressure (DBP) in the supine and standing positions are shown as mean and standard deviation in mmHg. In subgroups with count n=1, the result is in brackets as valid statistics are not possible here. For the Orthostatic Hypotension diagnosis, the decrease to the minimal value standing is crucial; therefore, the minimal value is also given.

Orthostatic Subgroup	HOI	PST	**SBP supine**	**SBP standing**	**DBP supine**	**DBP standing**
**HOI only**	Yes	Regular	-	-	-	-
**Physiological**	No	Regular	108.7 ±9.0	109 ±9.6	59.3 ±2.7	62.9 ±5.3
**Definite PoTS**	Yes	At least one PoTS-Limit exceeded, sustained HR pattern	119.8 ±8.8	121.3 ±10.3	61.9 ±.5.5	68.6 ±9.0
**Possible PoTS**	Yes	At least one PoTS-Limit exceeded, less clearly sustained HR pattern (e.g. fluctuating)	106.3 ±8.1	112.1 ±10.5	63.3 ±8.7	70.3 ±9.1
**False Positive**	No	PoTS-Limit exceeded (sustained or unsustained)	113 ±8.7	111.7 ±9.1	63.9 ±9.4	65.2 ±7
**HOI + OSw/oT**	Yes	Symptoms without Tachycardia	107.4 ±5.1	107.9 ±5.9	59.7 ±4.2	65.2 ±6.5
**OSw/oT w/o HOI**	No	Symptoms without Tachycardia	(123.2)	(121)	(66.6)	(70.2)
**HOI + OH**	Yes	Orthostatic Hypotension	(102.6)	(85, minimal value 66)	(56.8)	(49.5)

**HOI + VVS**	Yes	Vasovagal Syncope	-	-	-	-
**HOI + Other**	Yes	not identifiable	-	-	-	-
**OH w/o HOI**	No	Orthostatic Hypotension	-	-	-	-
**VVS w/o HOI**	No	Vasovagal Syncope	-	-	-	-
**Other w/o HOI**	No	not identifiable	(111.8)	(120.8)	(62.0)	(66.6)

Supplementary Table 2. Sensitivity and Specificity of different methods to distinguish PoTS from No PoTS – relative Limit. "Sustained HR increase" above the upright PoTS limit was examined differently. "60%" of upright HR values, HR values of the "last 2" minutes of standing, HR values of the "last 3" minutes of standing, the "mean of the last 3" minutes(1) or "at least two" HR values(2), and "at least one" value during standing(2). Methods including at least 3 minutes of standing time, could not be applied in one case due to shorter standing time. In that case, the corresponding method for 2 minutes was used, and the overall result is marked with *. "At least two": The CJC Statement requires another minute in standing if the elevation of HR was seen in the last minute(2). This could not be done in one case, as the test had to be aborted due to strong symptoms (marked with **).

Above relative Limit	60%	last two	last three	mean of the last three	at least two	at least one
**Sensitivity**	0.71	0.43	0.57*	0.86*	1.0**	1.0
**Specificity**	0.83	0.76	0.83*	0.65*	0.64**	0.44

Supplementary Table 3. Sensitivity and Specificity of different methods to distinguish PoTS from No PoTS – absolute Limit. "Sustained HR increase" above the upright PoTS limit was examined differently. "60%" of upright HR values, HR values of the "last two" minutes of standing, HR values of the "last three" minutes of standing, the "mean of the last three" minutes(1) or "at least two" HR values(2), and "at least one" value during standing(2). Methods including at least 3 minutes of standing time, could not be applied in one case due to shorter standing time. In that case, the corresponding method for 2 minutes was used, and the overall result is marked with *. "At least two": The CJC Statement requires another minute in standing if the elevation of HR was seen at the last minute(2). This could not be done in one case (different from above), as the test had to be aborted

due to strong symptoms (marked with **). Yet, this did not affect the result as that case also did not qualify for PoTS.

Above absolute limit	60%	last two	last 3	mean of the last three	at least two	at least one
**Sensitivity**	1.0	0.71	0.86*	1.0*	1.0**	1.0
**Specificity**	0.93	0.93	0.90*	0.83*	0.79**	0.73

1. Lee J, Vernon SD, Jeys P, Ali W, Campos A, Unutmaz D, et al. Hemodynamics during the 10-minute NASA Lean Test: evidence of circulatory decompensation in a subset of ME/CFS patients. J Transl Med. 2020;18(1):314.
2. Raj SR, Guzman JC, Harvey P, Richer L, Schondorf R, Seifer C, et al. Canadian Cardiovascular Society Position Statement on Postural Orthostatic Tachycardia Syndrome (POTS) and Related Disorders of Chronic Orthostatic Intolerance. Can J Cardiol. 2020;36(3):357-72.
